# Efficacy and Safety of Acetabular Cup without Screw Fixation in Total Hip Arthroplasty: A Systematic Review and Meta-Analysis

**DOI:** 10.3390/medicina58081058

**Published:** 2022-08-05

**Authors:** Takanori Miura, Hiroaki Kijima, Ryota Kimura, Jun Watanabe, Yuji Okazaki, Naohisa Miyakoshi

**Affiliations:** 1 Department of Orthopedic Surgery, Tazawako Hospital 17-1 Ukiyozaka Obonai, Tazawako, Senboku 014-1201, Akita, Japan; 2 Department of Orthopedic Surgery, Akita University Graduate School of Medicine, 1-1-1 Hondo, Akita 010-8543, Akita, Japan; 3 Scientific Research Workshop Peer Support Group (SRWS-PSG), Osaka, Osaka, Japan; 4 Department of Surgery, Division of Gastroenterological, General and Transplant Surgery, Jichi Medical University, 3311-1 Yakushiji, Shimotsuke City 329-0498, Tochigi, Japan; 5 Center for Community Medicine, Jichi Medical University, 3311-1 Yakushiji, Shimotsuke City 329-0498, Tochigi, Japan; 6 Department of Emergency Medicine, Hiroshima City Hiroshima Citizens Hospital, 7-33, Motomachi, Naka-ku, Hiroshima City 730-8518, Hiroshima, Japan

**Keywords:** bone screws, cementless acetabular cup, hip osteoarthritis, meta-analysis, systematic review, total hip replacement

## Abstract

*Background and Objectives*: Adequate initial fixation of the uncemented acetabular component in total hip arthroplasty is necessary to achieve long-term survival. Although screw fixation contributes to improved cup stability, there is currently no consensus on the use of this method. This study aimed to assess the existing randomized controlled trials (RCTs) on the efficacy and safety of cup fixation in total hip arthroplasty without screws. *Materials and Methods*: We searched the EMBASE, MEDLINE, Cochrane Central Register of Controlled Trials, World Health Organization International Clinical Trials Registry Platform, and ClinicalTrials.gov databases to identify RCTs published before February 2022. Primary outcomes were reoperation, cup migration, and Harris Hip Score. Secondary outcomes were the presence of a radiolucent line in the acetabular region, translation and rotation movement, and polyethylene wear. We conducted meta-analyses using the random-effects models. The revised Cochrane risk-of-bias tool was used to assess the risk of bias for outcomes of interest; the Grading of Recommendations, Assessment, Development, and Evaluation approach was used to summarize the body of evidence. *Results*: We included six reports from four studies. Total hip arthroplasty without screw fixation to the acetabular cup had little to no effect on reoperation (pooled relative risk, 0.98; 95% confidence interval, 0.14–6.68; *I*^2^ = 0%), cup migration (pooled relative risk, 1.72; 95% confidence interval, 0.29–10.33; *I*^2^ = 1%), Harris Hip Score (mean difference, 1.19; 95% confidence interval, −1.31–3.70; *I*^2^ = 0%), radiolucent line (pooled relative risk, 5.91; 95% confidence interval, 0.32–109.35), translation and rotation of all axes, and polyethylene wear (mean difference, 0.01; 95% confidence interval, −0.01–0.04; *I*^2^ = 0%), with very low certainty of evidence on all measures. *Conclusions*: The efficacy of acetabular cups without screw fixation in total hip arthroplasty remains uncertain, suggesting the need for prudent clinical application. Further large-scale, well-designed studies with low risk of bias are required.

## 1. Introduction

The number of total hip arthroplasty (THA) surgeries is set to increase because of the increasing elderly population and excellent outcomes of THA. Adequate fixation of the uncemented acetabular component is necessary to achieve bone ingrowth and ongrowth for successful long-term fixation [1]. The development of metal implants and surface treatments has improved the initial stability of cementless cups and resulted in their increased use, with good outcomes reported in recent years [2,3]. Although the use of a press-fit cementless cup is appropriate to achieve initial stability, there is a potential risk of cup migration and decreased survival rate if the stability is poor. Thus, the issue regarding the loosening and migration of the acetabular cup, which requires revision surgery, is yet to be resolved [4].

Additional screw fixation is often applied to improve acetabular cup stability during initial fixation [5]. Although it may not reduce the need for future revision or reoperation [6], there are reports of periacetabular osteolysis owing to the infiltration of joint fluid and polyethylene wear particles through the screw hole that may lead to loosening of the cup in the long term [5,7,8,9]. Therefore, the long-term effectiveness of screws in acetabular cup THA remains unclear.

Several randomized controlled trials (RCTs) [8,10,11,12,13,14] and a few systematic reviews [14,15,16] have investigated the effects of screw fixation in cementless cup THA. The first systematic review published by Ni et al. in 2014 [14] was updated by Fei et al. in 2020 [16] and Ni et al. in 2022 [17]. While these reviews integrated the existing data to provide clinically useful data and reported no benefits of screw fixation, the risk-of-bias assessment was inadequate. Moreover, the quality of evidence based on the Grading of Recommendations Assessment, Development, and Evaluation (GRADE) approach was not evaluated [18].

Because of the uncertainty of the results in previous studies, there is no clear consensus regarding the addition of screw fixation to the acetabular cup during THA. Therefore, this study aimed to summarize and critically appraise the existing RCTs that investigated the efficacy and safety of cup fixation without screws.

## 2. Materials and Methods

This review was conducted according to the reporting guidelines outlined by the Preferred Reporting Items for Systematic Reviews and Meta-analysis (PRISMA) [19]. Table S1. shows PRISMA 2020 checklist. (Table S1). This protocol was registered on the Open Science Framework (Available online: https://osf.io/59v2j/ (accessed on 4 February 2022)).

### 2.1. Eligibility Criteria

We included individual RCTs that assessed the efficacy and safety of acetabular cup fixation without screws in primary THA. No language or country restrictions were applied, and published and unpublished papers, conference abstracts, and letters were included. Furthermore, no studies were excluded on the basis of the observation period or year of publication. Cluster-randomized trials, crossover trials, and non-RCTs were excluded.

### 2.2. Participants

Adult patients (>18 years) who underwent primary THA with cementless cups due to primary or secondary hip osteoarthritis (OA) and acetabular or hip fractures, including unilateral or bilateral hip conditions, were included. No restrictions regarding the type of fixation method or surgical approach were applied. Studies that included children, adolescents, or participants undergoing revision hip arthroplasty or more than one intervention were excluded.

### 2.3. Interventions and Comparators

The intervention comprised THA that utilized press-fitted cups without screw fixation, while the control comprised THA that utilized press-fitted cups with fixation using one or more screws.

### 2.4. Outcome Measures

The following primary outcomes were measured: (1) the incidence of reoperation, which included any revision surgery of THA and other operations to relieve symptoms following THA during the follow-up period; (2) the migration of acetabular cup measured using radio-stereometric analysis (RSA) on radiographs and specialized software [20]. In RSA, translation is measured in the medial–lateral, distal–proximal, and anterior–posterior displacements, and rotation is measured in the transverse, longitudinal, and sagittal axes [20]. Furthermore, proximal migration of the cup up to 0.2 mm is considered acceptable, while proximal migration of the cup ≥ 1.0 mm is considered unacceptable [4]. Movement of >1.0 mm was defined as acetabular cup migration [4]; (3) the Harris Hip Score (HHS) used to evaluate patients following THA [21]. HHS is a physician-completed instrument that consists of subscales for pain severity (one item, 0–44 points), function (seven items, 0–47 points), absence of deformity (one item, 0–4 points), and range of motion (two items, 0–5 points). Scores of 0–70, 70–79, 80–89, and ≥90 points were defined as poor, fair, good, and excellent, respectively.

The secondary outcomes were as follows: (1) the presence of a postoperative radiolucent line >1.0 mm on radiographs in any acetabular regions [22]; (2) translational and rotational movement of the cup using RSA to measure the medial–lateral, distal–proximal, and anteroposterior translational distances and the rotation on the transverse, longitudinal, and sagittal axes [20]; (3) the polyethylene wear rate defined as a change (decrease) in postoperative polyethylene wear per year and calculated by the distance of the femoral head translation during follow-up divided by the follow-up period; (4) all adverse events reported by the original authors.

### 2.5. Search Strategy and Study Selection

We searched EMBASE via Dialog, MEDLINE via PubMed, the Cochrane Central Register of Controlled Trials (CENTRAL), the World Health Organization International Clinical Trials Registry Platform (ICTRP), and ClinicalTrials.gov on 5 February 2022 using specific keywords (Supplementary Material Table S2). We also scanned the reference lists of all studies, including international guidelines [23,24], and the reference lists of eligible studies. Two reviewers independently screened the titles and abstracts of all candidate studies identified by the search. Studies that were not related to our research question were excluded. Those articles extracted by the reviewers were included in the full-text review and assessed for eligibility. Any disagreements were resolved through discussion or by consulting a third reviewer if necessary.

### 2.6. Data Extraction and Quality Assessment

Two reviewers used a standardized data collection form, which was developed to extract information regarding the population characteristics, such as age, sex, and diagnosis, nature of the intervention and control, surgical approach, composition of hip prosthesis, the number of screws used, follow-up periods, and outcomes of interest from each included study. For studies with multiple groups according to material or fixation method, data from the screw insertion and non-insertion groups were pooled. If there were multiple observations at different timepoints for the same result, the result measured at 24 months was selected; if this was not possible, the result was selected at the longest follow-up period available. Studies with missing data were obtained from the authors where necessary, and if data could not be obtained from the authors, the studies were excluded. Two reviewers independently evaluated the risk of bias according to version 2 of the Cochrane risk-of-bias tool for randomized trials (RoB 2) [25]. Any disagreements were resolved by discussion with two reviewers or a third reviewer if necessary.

### 2.7. Measurement of Treatment Effect

For the binary variables, such as all-cause reoperation, migration of acetabular cup, and postoperative radiolucent line in the acetabular regions, we utilized random-effect models to calculate the relative risk (RR) with a 95% confidence interval (CI). Similarly, for continuous variables, such as HHS, translational and rotational movement of the cup, and polyethylene wear, we used random-effects models to calculate the mean difference (MD) with 95% CI. Adverse events were summarized according to their definition in the original article and were not included in the meta-analysis.

### 2.8. Data Analysis and Synthesis

For outcomes amenable to meta-analysis, we used the Review Manager software (RevMan 5.4; The Nordic Cochrane Centre, The Cochrane Collaboration, Copenhagen, Denmark) to calculate the pooled summary estimates and generate forest plots. Where possible, we performed an intention-to-treat analysis for all dichotomous data associated with missing data. Following the Cochrane Handbook, we did not impute missing continuous data [26]. For the studies that did not report standard deviation (SD), SD was calculated using CI according to the Cochrane Handbook [26]. Statistical heterogeneity was assessed by visually checking forest plots and calculating the *I*^2^ statistic (*I*^2^-value: 0–40%, may not be important; 30–60%, may represent moderate heterogeneity; 50–90%, may represent substantial heterogeneity; 75–100%, presents considerable heterogeneity). If substantial heterogeneity (*I*^2^ > 50%) was present, the reason for heterogeneity was assessed with the Cochrane Chi-square test (Q test), and a *p*-value <0.10 was defined as statistically significant [26]. To assess reporting bias, we searched clinical trial registration systems (ClinicalTrials.gov and ICTRP) to conduct an extensive literature search for unpublished trials. Funnel plot analysis was not performed because <10 studies were included in the pooled analysis [26]. A random-effects model was selected for all analyses as it can account for variation across studies [26].

### 2.9. Certainty of Evidence

We tabulated the body of evidence included in this review, including the certainty of the evidence. The certainty of the evidence was assessed for each outcome prespecified in the protocol according to the GRADE approach [18].

### 2.10. Sensitivity Analysis

Sensitivity analyses were planned to detect potential heterogeneity and determine whether the level of risk of bias affected the effect estimates. The following prespecified sensitivity analyses of the primary outcomes were planned: (1) including only those participants who completed the study with complete data; (2) considering measurement period adjustment with changes in acetabular cup migration, translation, and rotation over the time measured at 24 months.

### 2.11. Difference between Protocol and Review

Subgroup analysis was not performed in this study because of insufficient data. Sensitivity analyses including only those participants with complete data were not performed because no studies contained incomplete data.

## 3. Results

### 3.1. Study Selection

**Figure 1:**
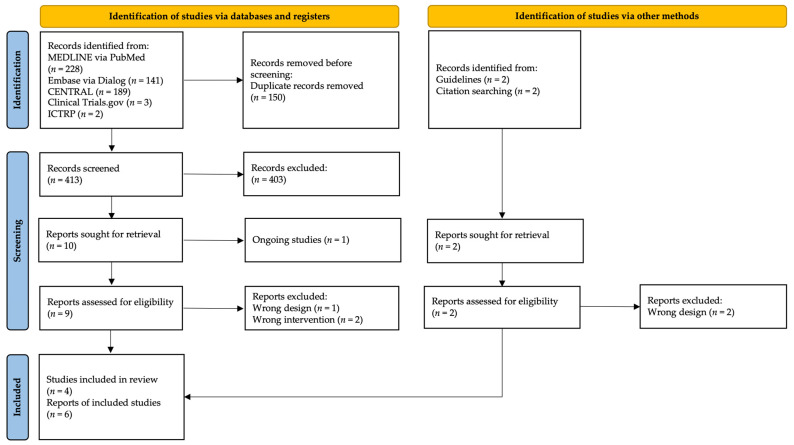
PRISMA flow diagram of the literature results. (CENTRAL: the Cochrane Central Register of Controlled Trials; ICTRP: the World Health Organization International Clinical Trials Registry Platform).

**Figure 2:**
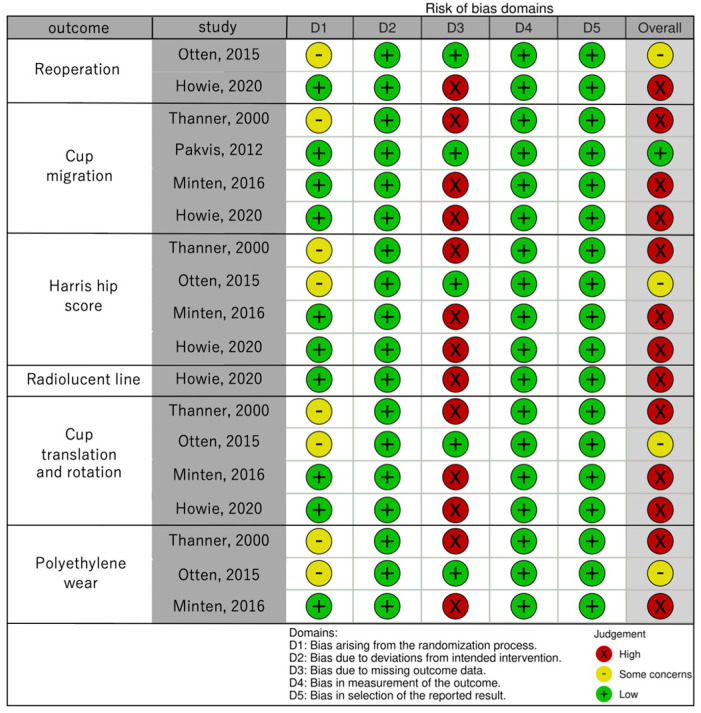
Risk-of-bias summary [8,10,11,12,13,14].

Figure 1 presents the study selection process. After removing duplicate studies, we screened 413 abstracts to identify 12 eligible studies for full-text screening. After screening, one study was excluded because of ongoing studies, three were excluded because of incorrect study design, and two were excluded because of incorrect intervention (Supplementary Material Table S3). Finally, six reports from four studies were included in this review. Table 1 summarizes the six RCTs (four studies) that compared the effects of screw insertion into press-fitted cementless cups. The risk of bias for quantitative synthesis is presented in Figure 2.

### 3.2. Primary Outcomes

#### 3.2.1. Reoperation

**Figure 3:**
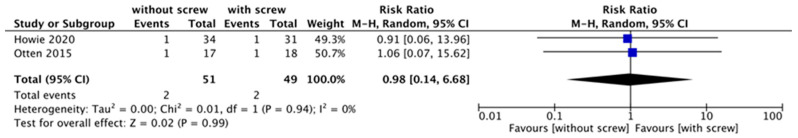
Forest plot of reoperation in the without and with screw fixation groups [10,14]. (M-H: Mantel-Haenszel test, df: degrees of freedom).

Four RCTs described reoperation [10,11,13,14]. Two reported no occurrence of reoperation [11,13], and the other two included one case of reoperation each in the with and without screw fixation groups. A meta-analysis was carried out using these two RCTs (Table 2; Figure 3) [10,14]. There was a high level of evidence uncertainty regarding the effect of acetabular cup THA without screw fixation on the outcome of reoperation (RR, 0.98; 95% CI, 0.14–6.68; *I*^2^ = 0%; very low certainty of evidence).

#### 3.2.2. Cup Migration

**Figure 4:**
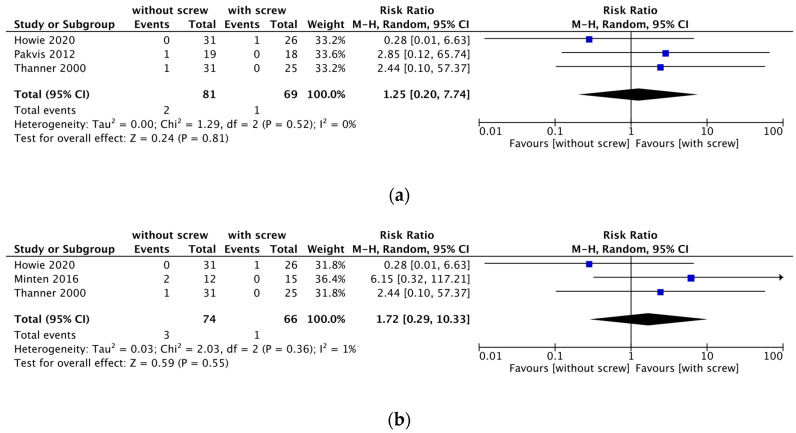
(**a**) Forest plot of acetabular cup migration with and without screw fixation. (**b**) Forest plot for sensitivity analysis for the cup migration [10,11,12,13].

A meta-analysis was carried out by summarizing data from three RCTs that measured the rate of cup migration (Table 2; Figure 4a) [10,11,13]. There was a high level of evidence uncertainty regarding the effect of no screw fixation on cup migration (RR 1.72; 95% CI, 0.29–10.33; *I*^2^ = 1%; very low certainty of evidence). We included three studies in the sensitivity analysis at 24 months (Figure 4b) [10,12,13]. The results of sensitivity analysis of cup migration at 24 months were consistent with the main results.

#### 3.2.3. HHS

**Figure 5:**
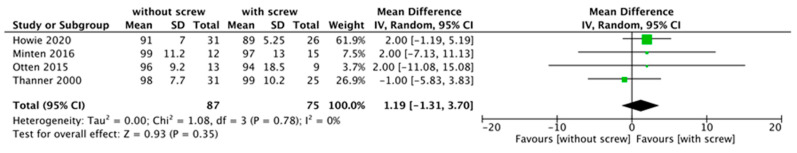
Forest plot of Harris Hip Score in the without and with screw fixation groups [10,11,13,14]. (IV: inverse variance method).

A meta-analysis was carried out by summarizing data from four RCTs that measured the HHS (Table 2; Figure 5) [10,11,13,14]. No screw fixation of the cup may have little to no effect on the HHS, but the evidence was considered low certainty (MD, 1.19; 95% CI, −1.31–3.70; *I*^2^ = 0%; very low certainty of evidence).

### 3.3. Secondary Outcomes

#### 3.3.1. Radiolucent Line in Acetabular Regions

Only one study described radiolucent lines in the acetabular regions (Table 2) [10]. The evidence suggests that the fixation of cups without screws results in no difference in the presence of radiolucent lines in the acetabular region (RR, 5.91; 95% CI, 0.32–109.35; very low certainty of evidence). Statistical heterogeneity was not applicable.

#### 3.3.2. Translation and Rotation

**Figure 6:**
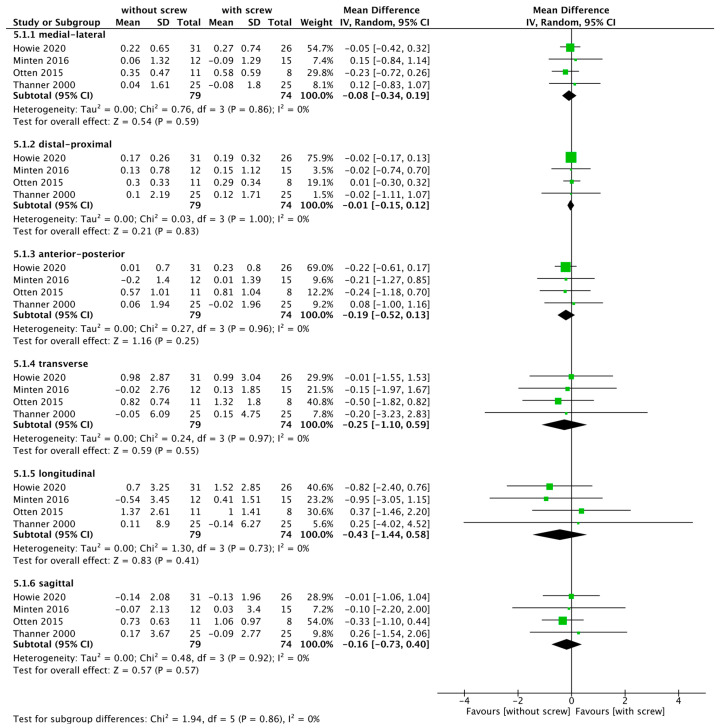
Forest plot of translation and rotation cup movement between the without and with screw fixation groups [10,11,13,14].

A meta-analysis was carried out by summarizing data from four RCTs that measured translational and rotational cup movement according to the RSA method (Table 2; Figure 6) [10,11,13,14]. There was a high level of evidence uncertainty regarding the effect of fixation without screws on medial–lateral (MD, −0.08; 95% CI, −0.34–0.19; *I*^2^ = 0%), distal–proximal (MD, −0.01; 95% CI, −0.15–0.12; *I*^2^ = 0%), and anteroposterior (MD, −0.19; 95% CI, −0.52–0.13; *I*^2^ = 0%) translational distances, as well as the effect of the transverse (MD, −0.25; 95% CI, −1.10–0.59, *I*^2^ = 0%), longitudinal (MD −0.43, 95% CI, −1.44–0.58; *I*^2^ = 0%), and sagittal rotational movements (MD, −0.16; 95% CI; −0.73–0.40; *I*^2^ = 0%). We included three reports in the sensitivity analysis due to adjustment measurement period at 24 months (Supplementary Material Figure S1) [10,12,13]. The results of the sensitivity analysis were consistent with the original results.

#### 3.3.3. Polyethylene Wear

**Figure 7:**
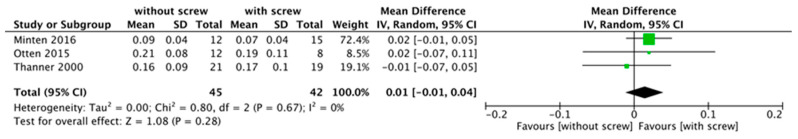
Forest plot of polyethylene wear between the without and with screw fixation groups [11,13,14].

A meta-analysis was carried out by summarizing data from three RCTs that measured polyethylene wear (Table 2; Figure 7) [11,13,14]. The evidence was very uncertain regarding the effect of no screw fixation on the outcome (MD, 0.01, 95% CI −0.01–0.04, *I*^2^ = 0%; very low certainty of evidence).

### 3.4. All Adverse Events

In the studies we included in our review, adverse events were reported in three reports (141 participants) [8,13,14]. Four participants (5.6%) in the without screw fixation group and six (8.7%) in the screw fixation group died during the follow-up period. Röhrl et al. reported one case of peroneal nerve palsy, two of dislocated hips, and one of femoral osteolysis, although the group in which they occurred remained unclear [8].

## 4. Discussion

### 4.1. Summary of Main Results

The present review, including four RCTs (six reports) and 183 participants, showed that acetabular cups without screw fixation might have little effect on revision surgery, cup migration, HHS, presence of a radiolucent line in the acetabulum, cup translation and rotation, and polyethylene wear compared to those with screws. However, there is very low certainty and insufficient evidence to conclude that acetabular cup THA without screws was effective and safe compared to that with screw fixation.

Although the measured outcomes showed no significant difference between the groups with and without the addition of screws in acetabular cup, in line with previous reviews [15,16,17], we concluded that the efficacy of acetabular cups without screw fixation remained uncertain, suggesting the need for prudent clinical application. Our conclusion was different from those of previous reviews. This could be attributed to the inclusion of only RCTs (to eliminate duplication of participants), evaluation of the risk of bias using RoB 2, and quality of evidence using the GRADE approach according to the Cochrane guidelines [19]. In the two previous systematic reviews, there was methodological concern regarding observational studies and RCTs being judged separately, and the effect size of these studies with different study designs was synthesized together. Subsequently, an earlier systematic review that synthesized the effect size from observational studies and RCTs separately reported that it was unnecessary to add screws to the acetabular cup [17]. However, duplication of participants was assumed because the same studies with different observation periods were synthesized in the osteolysis section. Our systematic review is methodologically sound in that it only included RCTs with strict study inclusion criteria, and it evaluated RoB and certainty of evidence. Further trials are needed to verify the effect of cups without adding screw fixation.

For clinical application, heterogeneity among studies included in this review should be considered. First, there were differences in acetabular cups. In cementless acetabular cup designs, porous surface coatings, which enhance implant stability, and titanium cups have demonstrated excellent results [27]. Furthermore, over the past two decades, tantalum cups with a higher coefficient of friction against bone and a unique metallic strut design resembling trabecular bone have been used, with reports of excellent fixation, osseous ingrowth, and a good clinical outcome similar to that of titanium cups [3,28]. In our included studies, titanium cups were used in three studies and tantalum cups were only used in one study in the group without additional screw fixation [10,11,13,14]. Second, the diagnoses of patients who underwent THA should be considered. Developmental dysplasia of the hip (DDH) is the leading cause of secondary hip osteoarthritis, with THA in these patients generally considered more complicated because of the pathomorphological changes in the acetabulum and femur [29]. While the results of press-fitted cementless cups in patients with DDH have been reported to be successful, various factors may affect cup fixation, such as acetabulum morphology, coverage of the cup by the bone, and cup placement position, with some reports of poor results [30,31].

Therefore, patient analysis with secondary OA is desirable to verify the efficacy of acetabular cup THA without screw fixation. While three studies included primary and secondary OA in the present review, they did not classify the results by diagnosis, and subgroup analysis could not be conducted [8,10,13,14]. Lastly, considering the variation in observation periods (range, 2–14 years) and the fact that we conducted sensitivity analysis at the 2 year timepoint, a longer follow-up time point analysis was impossible because of the lack of data. Therefore, these factors may limit the internal validity of the present results. Further subgroup analysis is warranted to improve clinical generalizability.

### 4.2. Strengths

The strength of this systematic review and meta-analysis was that we included only RCTs reporting the effect of the acetabular cup without screw fixation that evaluated outcomes of interest with the quality of evidence using the GRADE approach. Furthermore, we carefully and rigorously designed the screening, extraction, and scoring process according to the Cochrane Handbook [26].

### 4.3. Limitations

Our study had several limitations. Firstly, only four RCTs examining the effect of acetabular cups without screw fixation in THA were included, and the hip variation in the prosthesis materials, as well as the follow-up periods, made interpretation difficult. Secondly, the risk of bias in all included studies was either “high” or “of some concern”, and the certainty of the evidence was very low. Future trials with large-scale, well-designed trials with fewer missing data and lower risk of bias are required. Lastly, although subgroup analysis was planned on the basis of the patient’s underlying diagnosis (primary or secondary OA), the analysis was not performed because of the lack of data regarding surgical outcomes for each patient’s underlying diagnosis.

## 5. Conclusions

This systematic review evaluated complications, such as reoperation and polyethylene wear and osteolysis, as well as the stability and function of cementless cups, without screw fixation compared to those of screw-fixated cementless cups. Our findings suggest a very low certainty of evidence regarding the efficacy of acetabular cups without screw fixation in THA to determine whether the intervention could be applied in clinical practice. Given the small number of studies and participants, the heterogeneity of the samples, and the significant variation in the prosthesis materials and the follow-up periods, further large-scale, well-designed RCTs with low risks of bias are required to present convincing conclusions concerning the efficacy and safety of acetabular cups without screw fixation.

## Figures and Tables

**Table 1:** Characteristics of included studies.

Authors [Ref. No.]	Year	Country	Number of Hips (No Screw/Screw)	Age (SD; Years) (No Screw/Screw)	Surgical Approach	Materials of Acetabular Cup	Femoral Stem/Femoral Head/Liner	Follow-Up Period (Years)
Thanner et al. [13]	2000	Sweden	34/30	56 (10.8)/56 (10.8)	Modified Hardinge	Titanium fiber–metal cup	Uncemented; cemented/ceramic; cobalt-chrome/PE	2
Otten et al. [8,14]	2015	Sweden	17/17	55 (6.5)/56 (6.3)	Posterolateral	Porous-coated titanium alloy cup	Cemented; cementless/ceramic/PE	14
Minten et al. [11,12]	2016	The Netherlands	19/17	69 (8.4)/70 (5.5)	Posterolateral	All polyethylene, press-fit cup with titanium coating	Cementless/ceramic/PE	6.5
Howie et al. [10]	2020	Australia	35/31	58 (5.8)/57 (5.3)	Posterior	Tantalum trabecular metal (no screw)/titanium fiber-metal (screw)	Cemented/cobalt-chrome/PE	2

PE, polyethylene.

**Table 2:** Summary of findings.

Outcomes	No. of Participants (No. of RCTs)	Certainty of the Evidence (GRADE)	RR (95% CI)	Anticipated Absolute Effects	
				Risk with Screw	Risk Difference without Screw
Reoperation	183 (4)	⨁◯◯◯^*^ Very low ^a,b,c^	0.98 (0.14–6.68)	22 per 1000	0 fewer per 1000 (19 fewer to 128 more)
Migration of acetabular cup	140 (3)	⨁◯◯◯ Very low ^a,b,c^	1.72 (0.29–10.33)	15 per 1000	11 more per 1000 (11 fewer to 141 more)
Harris Hip Score	162 (4)	⨁◯◯◯ Very low ^a,b,c^		Mean range, 89–99	MD 1.19 higher (1.31 lower to 3.7 higher)
Radiolucent line	57 (1)	⨁◯◯◯ Very low ^a,b,c^	5.91 (0.32–109.35)	0 per 1000	0 fewer per 1000 (0 fewer to 0 fewer)
Cup translation: medial–lateral	153 (4)	⨁◯◯◯ Very low ^a,b,c^		Mean range, −0.09–0.58	MD 0.08 lower (0.34 lower to 0.19 higher)
distal–proximal	153 (4)	⨁◯◯◯ Very low ^a,b,c^		Mean range, 0.12–0.29	MD 0.01 lower (0.15 lower to 0.12 higher)
anterior–posterior	153 (4)	⨁◯◯◯ Very low ^a,b,c^		Mean range, −0.02–0.81	MD 0.19 lower (0.52 lower to 0.13 higher)
transverse	153 (4)	⨁◯◯◯ Very low ^a,b,c^		Mean range, 0.13–1.32	MD 0.25 lower (1.1 lower to 0.59 higher)
longitudinal	153 (4)	⨁◯◯◯ Very low ^a,b,c^		Mean range, −0.14–1.52	MD 0.43 lower (1.44 lower to 0.58 higher)
sagittal	153 (4)	⨁◯◯◯ Very low ^a,b,c^		Mean range, −0.13–1.06	MD 0.16 lower (0.73 lower to 0.4 higher)
Polyethylene wear	87 (3)	⨁◯◯◯ Very low ^a,b,c^		Mean range, 0.07–0.19	MD 0.01 higher (0.01 lower to 0.04 higher)

The risk in the intervention group (and its 95% CI) is based on the assumed risk in the comparison group and the relative effect of the intervention (and its 95% CI). ^*^ mean very low certainty of evidence. RCTs, randomized controlled trials; GRADE, Grading of Recommendations Assessment, Development, and Evaluation; CI, confidence interval; RR, risk ratio; MD; mean difference. GRADE Working Group grades of evidence: high certainty: we are very confident that the true effect lies close to that of the estimate of the effect; moderate certainty: we are moderately confident in the effect estimate, and the true effect is likely to be close to the estimate of the effect, but there is a possibility that it is substantially different; low certainty: our confidence in the effect estimate is limited, and the true effect may differ substantially from the effect’s estimate; very low certainty: we have very little confidence in the effect estimate, and the true effect is likely to be substantially different from the estimate of effect. ^a^ Downgraded one level due to serious risk of bias; the intervention methods were complex and missing outcome data. ^b^ Downgraded one level due to serious imprecision; the sample size was small. The sample size did not meet the criteria of the optimal information size (OIS; 400). OIS was 400 if alpha D = 0.05, beta D = 0.2, delta D = 0.2. ^c^ Downgraded one level due to serious imprecision; the CI crossed no difference.

## Data Availability

All data and materials can be retrieved from the references and articles included in the systematic review.

## Supplementary Materials

The following supporting information can be downloaded at https://www.mdpi.com/article/10.3390/medicina58081058/s1: Figure S1. Forest plot for sensitivity analysis for the translation and rotation movement of the cup; Table S1. PRISMA 2020 checklist; Table S2. Search strategies; Table S3. Reasons for exclusion of six reports.
